# Odd-Mode Surface Plasmon Polaritons Supported by Complementary Plasmonic Metamaterial

**DOI:** 10.1038/srep09250

**Published:** 2015-03-18

**Authors:** Xi Gao, Liang Zhou, Tie Jun Cui

**Affiliations:** 1 School of Information and Communication, Guilin University of Electronic Technology, Guilin 541004, China; 2 State Key Laboratory of Millimeter Waves, Department of Radio Engineering, Southeast University, Nanjing 210096, China

## Abstract

Surface plasmon polaritons (SPPs), either on metal-dielectric interfaces in optical frequencies or on structured metal surfaces in the lower frequencies, are dominantly even modes. Here we discover dominant odd-mode SPPs on a complementary plasmonic metamaterial, which is constructed by complementary symmetric grooves. We show that the fundamental SPP mode on such a plasmonic metamaterial is a tightly confined odd mode, whose dispersion curve can be tuned by the shape of groove. According to the electric field distributions of odd-mode SPPs, we propose a high-efficiency transducer using asymmetric coplanar waveguide and slot line to excite the odd-mode SPPs. Numerical simulations and experimental results validate the high-efficiency excitation and excellent propagation performance of odd-mode SPPs on the complementary plasmonic waveguides in the microwave frequencies.

The recent interest in the field of plasmonics is mainly due to the ability to spatially confine the electromagnetic energy in subwavelength scale at visible frequencies^1^. Surface plasmon polaritons (SPPs) on metal-dielectric interfaces open up a previously inaccessible length scale for the optical research, which implies the possibility to use SPPs for the miniature photonic circuits and interconnects^2^,^3^. The concept of highly localized wave guiding has also obvious advantages in the microwave and terahertz frequencies, where plasmonics enables high performance transmissions^4^,^5^, near-field imaging^6^, and sensing^7^,^8^. On the other hand, SPPs offer subwavelength field localizations only for frequencies close to the intrinsic plasma frequency of metal. For most metals, the plasma frequencies are located in the optical and even ultraviolet regions. At lower frequencies (microwave and terahertz), however, the metal behaves like a perfectly electric conductor (PEC), where the natural SPPs cannot be supported.

Plamsonic metamaterials formed by periodical textures on metal surfaces address the challenge of routing surface waves on subwavelength scale in microwave and terahertz frequencies^9^,^10^. Recently, the idea of tailoring the topography of metal to obtain the strong confinement to surface waves mimicking the behaviors of SPPs at optical frequencies has been studied in two-dimensional hole lattices^11^ and one-dimensional (1D) groove arrays^12^ decorated into flat metal surface or metal wires. Then these findings have been experimentally demonstrated in the microwave regime^13^. Due to their mimicking characteristics, the surface waves supported by textured metallic surface were termed as designer SPPs or spoof SPPs (SSPPs). The main advantage of SSPPs is that their dispersions can be tuned at will by shapes and dimensions of subwavelength holes or grooves machined into metal surface, providing an effective method to guide and manipulate electromagnetic waves in the form of SSPPs. Based on the attractive feature, many SSPP devices including filter, waveguide and frequency splitter were proposed^14^,^15^,^16^. Unfortunately, these early plasmonic devices have an obviously disadvantage of inherent three-dimensional geometrics, which rigorously limits their practical applications.

More recently, ultrathin corrugated metallic strips have been verified to guide the SSPP waves on planar paths^17^,^18^, and furthermore they can be bent, twisted, and wrapped to arbitrary surfaces to produce conformal surface plasmons (CSPs). Such ultrathin plasmonic metamaterials are good candidates for plasmonic integrated circuits in the microwave and terahertz frequencies^19^. Up to now, these ultrathin plasmonic metamaterials are dominantly formed by drilling one-sided grooves or symmetrically two-sided grooves on a metallic strip^20^,^21^, where even-mode SPP waves are dominantly confined, similar to the natural SPPs in the optical regime.

Here, we propose a complementary plasmonic metamaterial that supports dominantly odd modes, providing a very promising route to achieve anti-symmetrical subwavelength confinements. We show that the dispersion relations of SSPPs can be tuned by the shape of complementary symmetric grooves textured on the ultrathin metal plate. In such a structure, the fundamental odd-mode SSPPs act as the operating mode and the strong confinement to SSPPs appears in a wide frequency range. We demonstrate that the odd-mode SPPs can be sustained and exhibit a rich behavior in planar structures, including the 90° bending with very low loss and high-efficiency transducer of the odd-mode SPPs, which have been verified by experiments in the microwave frequencies.

## Results

### Design and dispersion relation of complementary plasmonic structures

**Figure 1:**
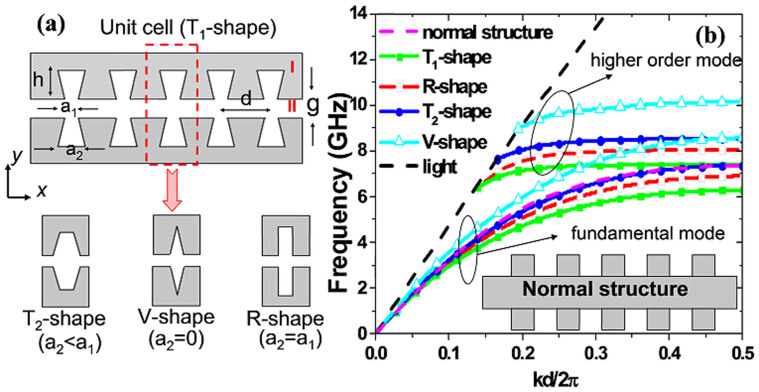
(a) Schematic diagram of the complementary plasmonic metamaterial. Region I is 0.018 mm-thick metal film supported by a dielectric plate with the thickness of 0.5 mm and dielectric constant of 3.38. Region II includes complementary symmetric grooves machined into the metal film. Four different shapes of grooves are considered: T_1_-shape (a_2_ > a_1_), R-shape (a_2_ = a_1_), T_2_-shape (a_2_ = 0), and V-shape (a_2_ < a_1_). (b) Dispersion relations of fundamental modes for the complementary plasmonic metamaterials with four kinds of groove-shapes and the normal structure with the rectangular groove. For comparison, the dispersion curves of higher modes are also displayed. The parameters are set as *d* = 5.5 mm, *h* = 6.5 mm, *g* = 1 mm, *a*_1_ = 1 mm, and *a*_2_ are chosen as four values (2 mm, 1 mm, 0.5 mm, and 0), respectively, to obtain T_1_-, R-, T_2_- and V-shaped grooves. The inset illustrates the normal structure with rectangular grooves, whose parameters are the same with the R-shaped groove in the complementary structure.

The schematic configuration of the complementary plasmonic metamaterial is depicted in Fig. 1(a). An array of complementary symmetric grooves of depth *h* and lattice constant *d* is machined into a metal film. Four kinds of groove shapes (marked as T_1_-, R-, T_2_- and V-shape) are provided for different values of *a*_2_. Using the finite element method, we analyze the dispersion relations of the surface electromagnetic waves supported by the structure. Under the PEC approximation, the frequencies of the dispersion bands are scaled with the reciprocal size of the structure. Therefore, the unit cell with length *d* is used. It is interesting to calculate the dispersion curves of transverse-magnetic polarized waves propagating along the *x*-direction (TM_x) with the axial propagation constant *k*. Fig. 1(b) shows the calculated dispersion relations, which include two branches corresponding to the fundamental and higher-order modes for different groove shapes, changing from the T_1_-shape to the V-shape. All bands deviate significantly from the light line, and this departure is greater when the groove changes from the V shape to T_1_ shape.

We remark that such dispersion curves exhibited similar behaviors of SPPs propagating along the metal-dielectric interfaces in the optical frequencies or the structured metal strips in the lower frequencies, presenting asymptotic frequencies. The asymptotic frequency is tuned by the shape of grooves, and the maximum value of tuning reaches to 2.5 GHz when we alter the shape of groove from T_1_ to V. For a 1D array of grooves^12^, it behaves as an anisotropic medium with *ε_x_* = *d*/*a* (*a* is width of rectangular groove) and *ε_y_* = *ε_z_* = ∞. In this case, the amendment of groove shape is equivalent to change *ε_x_*, resulting in the change of asymptotic frequency. For comparison, the dispersion relation of the normal structure (see the inset) with rectangular grooves is also calculated, as shown in the pink dashed line in Fig. 1(b). We observe that the asymptotic frequency of the complementary structure (the red line) is lower than that of the normal structure for the same groove shape. On the other hand, the dispersion relation of the complementary structure has a larger propagation constant than that of the normal structure at a fixed frequency, which implies that the complementary structure has stronger confinement ability to SSPPs wave.

### Mode characteristics and excitation of odd-mode SPPs

**Figure 2:**
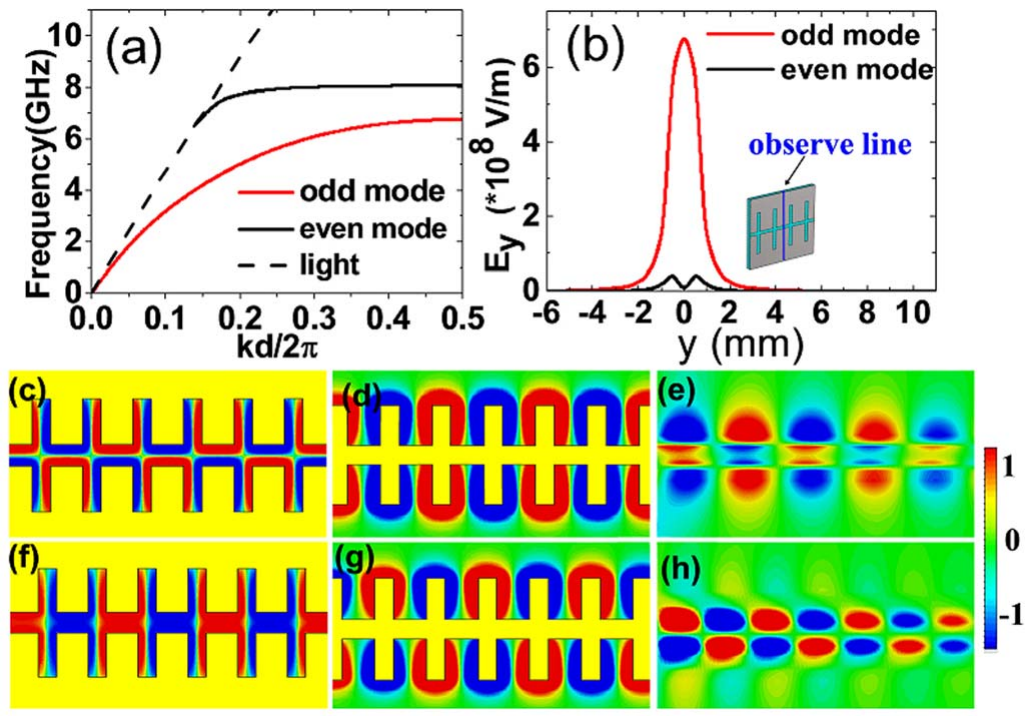
(a) Dispersion relations of complementary R-shaped grooves for odd mode and even mode. (b) The *E*_y_ field distributions for the two modes along the observation line shown in the inset. (c) The spatial variation of the *z*-component electric field (*E*_z_) for the dominant odd mode of the complementary plasmonic structure at 6.8 GHz. (d) The *E*_z_ field distribution for the dominant even mode of the normal corrugated metal strip at 7 GHz. (e) The *E*_z_ field distribution for the dominant even mode of the DMD interface in the optical regime at 300 THz. (f–h) The *E*_z_ field distributions for the high-order modes of the complementary structure (8 GHz), normal structure (7.8 GHz), and DMD interface (560 THz). The operating frequencies are all close to their asymptotic frequencies, and the yellow parts in Figure (c), (d), (f), and (g) indicate metal.

The complementary symmetric grooves can be looked as two 1D arrays of grooves spaced by a distance *g*, as shown in Fig. 1(a). For the two closely-packed 1D arrays of grooves (*g* is small), the SPP waves associated with each individual 1D array become coupled and hybridized into two surface modes. As a result, there are two dispersion relations corresponding to the two modes, as illustrated in Fig. 2(a). As expected, this complementary symmetric groove leads to two confined SPP modes with the lower frequency (the first mode) occupying a wider bandwidth, whereas the higher frequency (the second mode) with a narrow bandwidth, which is closer to the light line. In order to check the characteristics of the two SSPPs modes, we have conducted numerical simulations of the electric (*E*) field associated to SSPPs. Fig. 2(b) displays the variation of the *y*-component electric field (*E*_y_) for the two surface modes along the observation line in the inset. For the first mode, the *E*_y_ field is highly concentrated on the surface of the complementary symmetric grooves and the maximum *E*_y_ field appears at the centric position. It is noteworthy that the *E*_y_ field for the second mode is completely different. There are two peaks of *E*_y_ field and the two peaks emerge near the surface of each 1D array of grooves, instead of the central location. This feature makes it advantageous for excitation of the first mode in the following SPP device.

A key characteristic of such SSPP waves appears that the fundamental SPP mode is anti-symmetric (i.e., the odd mode), which is contrary to the natural SPPs in the optical frequency and SSPPs on normal plasmonic metamaterials. This property is clearly illustrated in Fig. 2(c), which gives the *E*_z_-field simulated in the *xy* plane above the complementary structure. We observe that the *E*_z_-field is anti-symmetric for the first mode (odd mode), whereas the second mode is symmetric (even mode) in higher frequencies, as shown in Fig. 2(f). This is obviously different from the phenomena of normal SSPP structures and the optical dielectric-metal-dielectric (DMD) interfaces, where the even mode is the fundamental mode. As comparisons, Figs. 2(d) and (e) provide the *E_z_*-field distributions for the normal SSPP structure and DMD interface, which have symmetric patterns, implying the even fundamental modes. Similarly, the high-order modes on the normal SSPP structure and DMD interface are illustrated in Figs. 2(g) and (h), showing the odd-mode properties. Actually, the proposed complementary plasmonic structure can be looked as a three-layer system (medium-air-medium) based on the equivalent medium theory. Whereas in the three-layer system, the odd-mode SPP wave is the fundamental mode^27^, which implies that the dominant odd-mode SSPPs is supported in the complementary plasmonic waveguide.

The attractive feature of the dominant odd-mode SSPPs in the complementary structure is the strong confinement ability and high transmission efficiency in broadband. For the normal plasmonic structure, it may exhibit weak confinement to SSPP waves when the SSPP propagation constant (*k*) is close to *k*_0_ at the light line, which makes the SSPP waves extend over many wavelengths into free space^27^. Therefore, the transmission efficiency of SSPP waves in the region of small *k* will be decreased. The same phenomenon is occurred in the optical region for SPPs in the DMD plasmonic waveguide. In general, the SPP waves for small *k* behave as the Sommerfeld-Zenneck waves^26^,^27^, and they usually occupy a wider frequency range in the dispersion relation, which affect the operation bandwidth of SSPPs/SPPs in normal plasmonic structures and DMD plasmonic waveguides. In the complementary grooves, however, the SSPP penetration depth in air is significantly reduced due to the narrow silt (*g* is small) and metal boundary on both sides. Especially, the Sommerfeld-Zenneck waves (with small *k*) can also be affectively confined into the narrow slit, which effectively increases the operating bandwidth of SSPPs.

**Figure 3:**
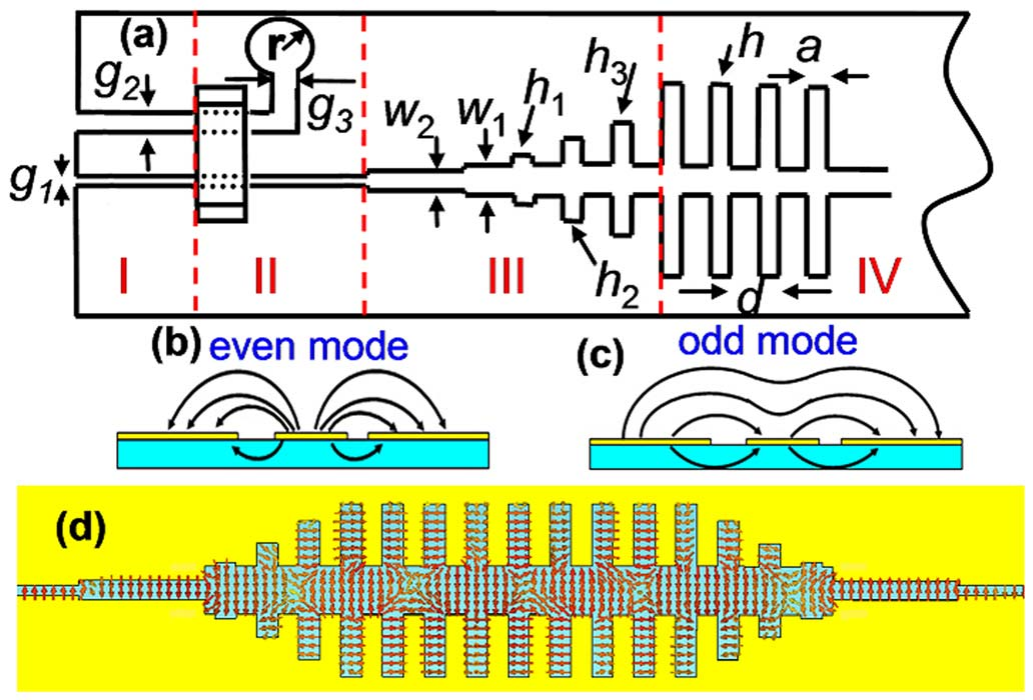
(a) Schematic picture of odd-mode SPP transitions from an asymmetric coplanar waveguide to the complementary plasmonic waveguide, in which *g*_1_ = 0.15 mm and *g*_2_ = 0.4 mm are selected in Region I to feed electromagnetic energies from the SMA connector. Region II is used to realize the mode conversion between the asymmetric coplanar waveguide and the slot line, in which an air bridge is employed to suppress the odd mode, and a circular balun with *r* = 5 mm and *g*_3_ = 1 mm is used to absorb the electromagnetic fields in the wider slot. Region III includes the stepped slot lines and gradient grooves, matching the impedance and wave vectors in broadband between the front slot line and SSPP waveguide. Region IV is the complementary plasmonic waveguide, in which the parameters are chosen as *w*_1_ = 1 mm, *w*_2_ = 0.4 mm, *h*_1_ = 0.5 mm, *h*_2_ = 2.4 mm, *h*_3_ = 4.7 mm, *h* = 6.5 mm, *d* = 5.5 mm, and *a* = 1 mm. (b) and (c) The distributions of electric fields for the even and odd modes in the asymmetric coplanar waveguide. (d) The electric-force lines in the complementary plasmonic waveguide.

The strong confinement of complementary symmetric grooves to the odd-mode SSPPs in broadband is advantageous for applications in plasmonic devices (e.g. waveguide and filter) with low loss, whereas a high-efficiency excitation of the odd-mode SSPPs is vital. The facts that the dispersion relation of SSPPs can be tuned by engineering the geometry of complementary grooves and the *E* field distribution is odd mode give us a clue to obtain a high-performance SSPP converter. Fig. 3(a) gives the schematic picture of the converter from guided waves to SSPPs. The gradient grooves with depths changing from *h*_1_ to *h*_3_^20^,^22^,^23^ shown in region III in Fig. 3(a) realize the gradual increase of wave vectors from the fast waves in the slot line to the slow waves (SSPPs) in the plasmonic waveguide, thus obtaining the perfect wave-vector matching. Asymmetric coplanar waveguide makes it convenient to manipulate its characteristic impedance at 50 Ω by altering *g*_2_ for fixed *g*_1_^24^. In this case, there are even mode and odd mode for the electric fields, as displayed in Figs. 3(b) and 3(c). The odd mode has high characteristic impedance (usually more than 60 Ω), which is difficult to match with the SMA connector. Therefore, an air bridge is employed to suppress the odd mode^25^. The balance balun in region II leads to the mode conversion from the asymmetric coplanar slot line. Then, the electromagnetic fields in the slot line effectively excite the odd-mode SSPPs in the complementary symmetric grooves due to their similar distributions of electric fields. Fig. 3(d) shows the conversion process of *E* field from slot line to complementary grooves, from which we clearly observe the field conversions.

### Straight and 90° bending plasmonic waveguide

**Figure 4:**
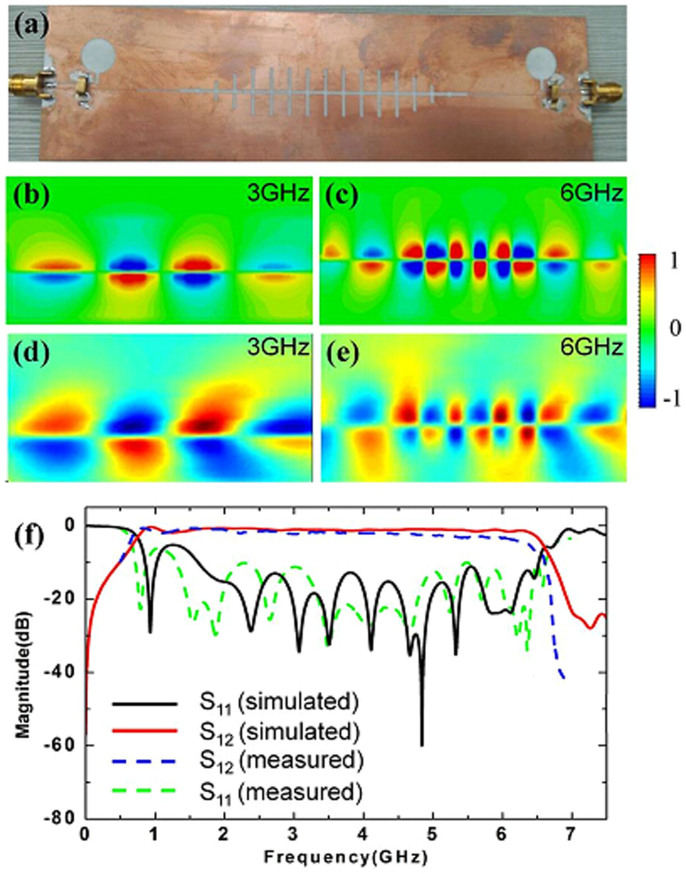
(a) The fabricated sample of the straight complementary plasmonic waveguide, including the conversion parts. (b)–(c) The simulated distributions of near electric fields (*E_z_* components) at two specific frequencies of 3 GHz and 6 GHz, in which 3 GHz is far away from the asymptotic frequency, whereas 6 GHz is close to the asymptotic frequency. (d)–(e) The measured distributions of near electric fields (*E_z_* components) at 3 GHz and 6 GHz. (f) The simulated and measured transmission coefficients (S_12_) and reflection coefficients (S_11_).

In order to confirm the broadband characteristics of complementary symmetric grooves, we have conducted numerical simulations and made experiments on a straight plasmonic waveguide and 90° bending based on the above excitations. The fabricated sample of straight plasmonic waveguide, including the conversion parts, is shown in Fig. 4(a). The simulated and measured distributions of near electric fields (*z*-components) at 3 and 6 GHz are illustrated in Figs. 4(b)–(e), respectively, showing very good agreements. As expected, the odd-mode SPPs waves are successfully excited and tightly confined by the plasmonic waveguide. The electric field at the input is nearly the same as that at the output, demonstrating very low transmission loss. Especially at 3 GHz, though its wave vector is close to the light line (see the red line in Fig. 2(a)), the field is still strongly bound by the complementary grooves. Fig. 4(f) shows the measured transmission and reflection coefficients (the blue and green dashed lines) for the straight waveguide. We also give the simulated results (the red and black solid lines) for comparisons, in which the insertion loss is less than −0.8 dB from 1.2 to 6.5 GHz. The good agreement between the simulations and experiments validates the high-efficiency excitation of odd-mode SPPs and low propagation loss in a broad frequency range.

**Figure 5:**
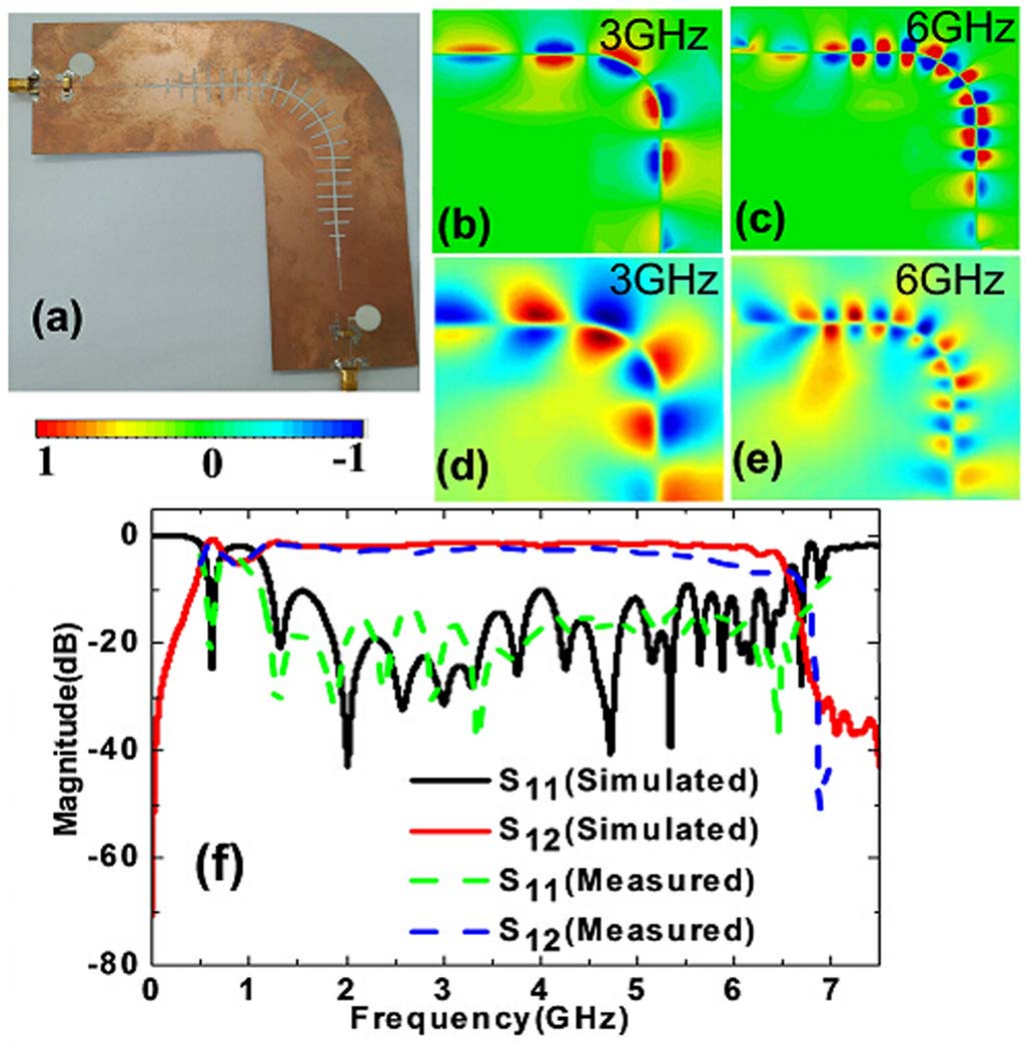
(a) The fabricated sample of the 90° bending plasmonic waveguide, including the conversion parts. (b)–(e) The simulated and measured distributions of near electric fields (*E_z_* components) at two specific frequencies of 3 GHz and 6 GHz. (f) The simulated and measured transmission coefficients (S_12_) and reflection coefficients (S_11_).

Furthermore, we also expect that the complementary grooves exhibit low loss when the odd-mode SPPs propagate along a bending path. Fig. 5 verifies this effect for a 90° bending waveguide with the radius of 31 mm (see Fig. 5(a)). From the simulated and measured near electric-field distributions shown in Figs. 5(b)–5(e), the odd-mode SPPs smoothly propagate along the circular arc with good modal shape and low attenuation. We remark again the high confinements of SPPs at the low frequency (see Figs. 5(b) and 5(d)). The transmission and reflection coefficients in presented in Fig. 5(f) demonstrate that the insertion loss is less than −1 dB in the frequency range from 1.2 to 6.5 GHz, implying excellent performance in wideband. The simulation results are in good agreements with the experiments except the little deviation at high frequencies. The deviation may be caused by the tolerance of fabrication, especially in the bending zone. The scaling-down geometry has great promise to realize high-efficiency plasmonic waveguide in the terahertz regime.

## Discussion

We have shown that the complementary symmetric grooves can sustain odd-mode SSPPs and their dispersions can be tuned by the shape of grooves. Apart from the good confinement for SSPPs with larger wave vector, the proposed structure can effectively bound the surface waves whose wave numbers are close to the light line, and hence results in the broadband performance. To excite the broadband plasmonic waveguide, we propose an asymmetric coplanar waveguide and slot line to convert the guided modes to the odd-mode SPPs. The simulation and experimental results of near electric fields and S parameters have demonstrated the efficient conversions and transmissions of the odd-mode SSPPs. Especially for the 90° bend, high performance is observed in wideband frequencies. We believe that these results pave a way to develop broadband odd-mode SPP functional devices and circuits in the microwave and terahertz regimes.

## Methods

### Simulation

With the help of commercial software, CST Microwave Studio, we study on the dispersion relations and surface fields of the complementary plasmonic metamaterials. In this case, the dispersion relations (*f-k* curve) are obtained by calculating the eigen frequencies in a unite cell and the distributions of surface field are simulated in a plasmonic waveguide with length of 6 unit cells by using the Eigenmode analysis.

### Sample fabrication

As shown in Figs. 4(a) and 5(a), the planar complementary plasmonic waveguides are fabricated on 0.508 *mm* thickness dielectric layer whose permittivity and loss tangent are 3.38 and 0.0027, respectively.

### Measurement

In experiments, the samples were put on the foams blocks. To get the S parameters, both the two ports of the straight and bent waveguides connected with a vector network analyzer respectively. The distributions of the surface electric fields along the plasmonic waveguide are mapped by a near-field scanning system. The experimental setup consists of an Agilent N5230C vector network analyzer, coaxial line, a wideband impedance matching device, and a monopole antenna as detector. The coaxial line is connected to one port of the plasmonic waveguide to feed the electromagnetic waves. The detector, controlled by two computer-controlled step motors, is fixed at 2 mm above the sample and moved to scan the *E_z_* fields. The scanning step is 1 mm. However, there is another port for the plasmonic waveguide. If the port is suspended, the EM waves will be totally reflected into the plasmonic waveguide, which will affect the distribution of surface fields. In order to eliminate the reflection, a wideband impedance matching device is used to connect the port.

## Author Contributions

X.G. and L.Z. proposed and designed the theory of Complementary Plasmonic waveguide, performed initial verification. T.J.C. supervised the design and experiments, and wrote the manuscript.
